# PERCEIVED MENTAL FATIGABILITY: NOVEL INSIGHTS INTO SOCIOBEHAVIORAL CORRELATES AND HERITABILITY

**DOI:** 10.1093/geroni/igz038.863

**Published:** 2019-11-08

**Authors:** Nancy W Glynn, Eleanor M Simonsick

**Affiliations:** 1 University of Pittsburgh, Department of Epidemiology, Pittsburgh, Pennsylvania, United States; 2 Longitudinal Studies Section, Intramural Research Program, National Institute on Aging, Baltimore, Maryland, United States

## Abstract

Fatigue, a common patient-reported outcome, is a unique risk factor associated with both cognitive and physical function. Perceived mental fatigability, a self-report measure of cognitive fatigue anchored to activities of fixed intensity and duration, eliminates self-pacing bias, and therefore is a more sensitive measure of the degree to which cognitive tiredness limits activity. Higher perceived mental fatigability has been associated with functional decline and lower grey matter brain volumes in older adults. We developed the Pittsburgh Fatigability Scale (PFS), a self-administered, 10-item tool to assess perceived physical and mental fatigability across a range of activities, which is widely used internationally. We previously validated the PFS physical subscale. Using a large multicenter international cohort, the Long Life Family Study, we will present the validation of the PFS mental subscale, examine its epidemiology, and explore genetic and socio-behavioral factors associated with perceived mental fatigability in older adults. Specifically, Ms. Renner will share the results of the validation of the PFS mental subscale; Ms. Meinhardt will present heritability and prevalence of higher perceived mental fatigability across age strata and sex; and Ms. Gmelin will consider the link between stress and coping styles on perceived mental fatigability. Further, using a smaller methodological study, the Developmental Epidemiologic Cohort Study, Ms. Graves will explore whether diurnal patterns of physical activity using accelerometry differ in older adults with higher versus lower perceived mental fatigability. Dr. Simonisick, our Discussant, will critically review the presentations and share future directions to inform potential interventions aimed at lowering perceived mental fatigability.

